# Inter-observer agreement in interpreting intraoperative ultrasonography during brain tumour surgery

**DOI:** 10.3389/fsurg.2025.1679617

**Published:** 2025-10-23

**Authors:** Aimun A. B. Jamjoom, Olivier J. J. Sluijters, Jack Wildman, Davide Giampiccolo, Constantinos Charalambides, Neil U. Barua

**Affiliations:** 1 Department of Neurosurgery, Queens Hospital, Romford, United Kingdom; 2 The Blizard Institute, Queen Mary University of London, London, United Kingdom; 3 Department of Neurosurgery, Southmead Hospital, Bristol, United Kingdom; 4 Department of Clinical and Experimental Epilepsy, Institute of Neurology, University College London, London, United Kingdom

**Keywords:** intra-operative ultrasonography, brain tumour surgery, observer agreement, machine learning, glioma

## Abstract

**Background:**

Intraoperative ultrasonography (iUS) is a powerful technology that is being increasingly utilized for brain tumour surgery. However, ultrasonography has been documented to be observer dependent in a range of healthcare settings. Here we objectively assess the degree of inter-observer variability in iUS for brain tumour surgery.

**Methods:**

Nine images taken from routinely collected iUS videos from brain tumour surgery were presented to 18 neurosurgeons (5 consultants, 7 senior fellows, 6 residents). This included three tumour types [metastasis, high-grade (HGG) and low-grade glioma (LGG)] at three operative stages (before, during and near resection completion). Using 3D Slicer, observers segmented what they deemed to be tumour. Digital Image Correlation Engine Similarity Coefficients (DSC) were calculated to examine inter-observer variability.

**Results:**

A total of 1,377 DSCs were calculated between 18 observers across 9 images. Metastasis had the highest DSC (0.72 ± 0.32), followed by HGG (0.64 ± 0.33) and LGG (0.58 ± 0.25; *p* < 0.00001). As the resection progressed, the degree of inter-observer agreement broke down. Before resection the DSC was 0.87 ± 0.11; during resection (0.74 ± 0.17) and at completion (0.32 ± 0.27; *p* < 0.00001). The trend of decreasing agreement as the resection progressed held across tumour types. Observers reported increasing difficulty with iUS interpretation as the resection proceeded and there was statistically significant (*p* = 0.014) negative correlation (−0.775) between DSC and difficulty rating of the segmentation.

**Conclusion:**

Here we demonstrate significant inter-observer variability in iUS for brain tumour surgery. The degree of variability is tumour-type and operative stage dependent. This work adds weight to the value of building machine learning augmented iUS for brain tumour surgery.

## Introduction

Intraoperative ultrasonography (iUS) is a powerful technology that is increasingly being utilized to support surgical resection of brain tumours. iUS has several benefits compared to other intra-operative imaging modalities such as intraoperative MRI including: real-time visualisation of the tumour, more readily available and cost efficient to implement. Importantly, it is also easily integrated into surgical work-flows and does not increase operative time as significantly as intra-operative MRI (1). Synthesis of the literature points towards iUS enhancing glioma extent of resection which is an important determinant of improved survival (2, 3). Though point-of-care ultrasonography (performed and interpreted by the treating clinician at bedside) is user-friendly, there are learning curves for the psychomotor skills required and image interpretation (4).

There is evidence of variable inter-observer agreement in interpreting ultrasound images from different organs including thyroid, pelvic, musculoskeletal and lung examinations (5–9). Interpreting iUS images in brain tumour surgery is complicated by posterior enhancement of the floor of the resection cavity (10). A study examining agreement across 30 brain tumour iUS images found moderate levels of inter-observer variance (11). Importantly, this study did not examine the impact of operative stage on segmenting iUS images. This is a limitation as the most difficult and important point to interpret iUS images and near the end of tumour resection when the surgeon is using ultrasound to help assist identifying residual tumour. Our study aims to evaluate the degree of inter-observer agreement between a group of neurosurgeons in interpreting iUS images from three different brain tumours and across three operative stages.

## Methods

**Figure 1:**
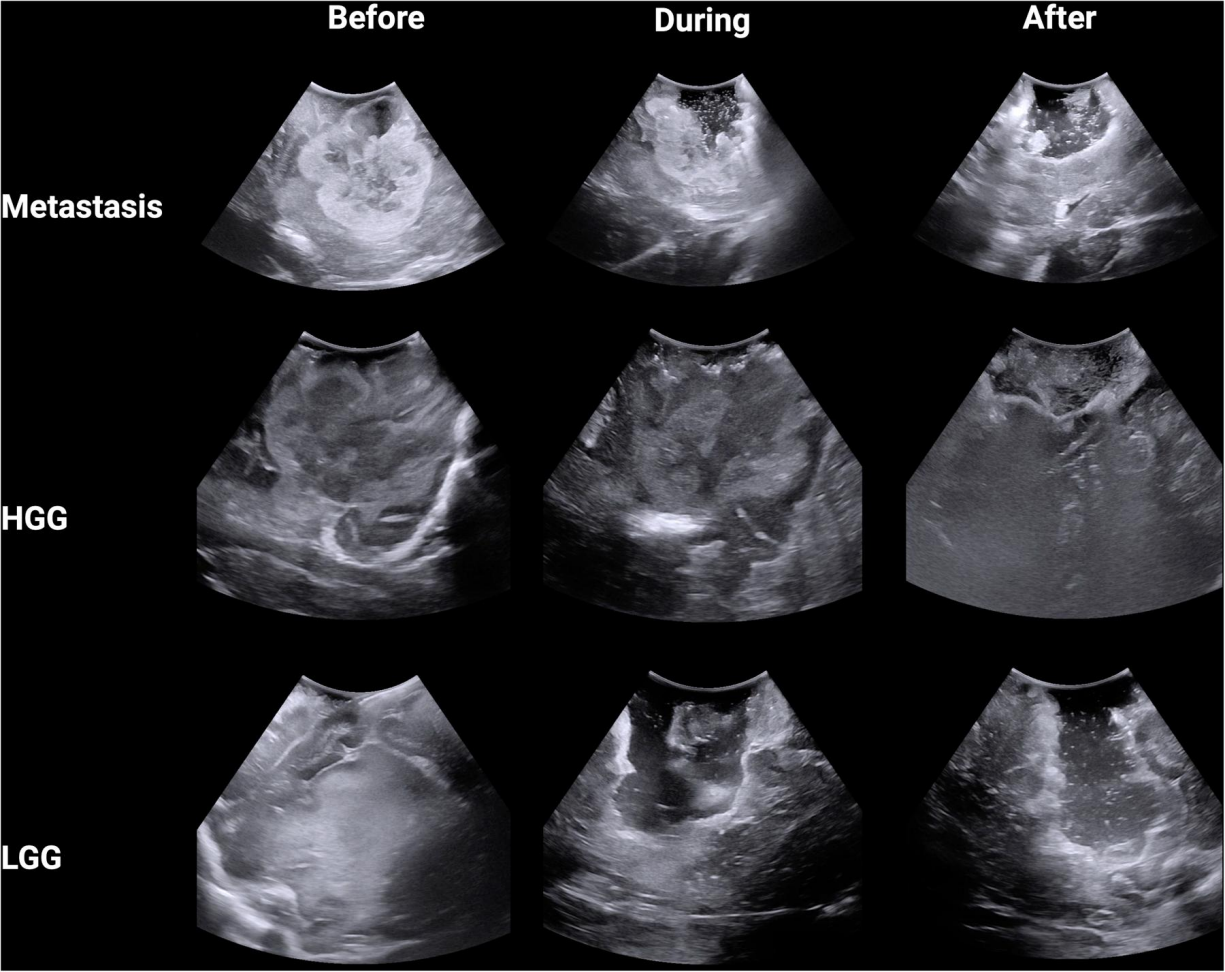
Intra-operative B-mode ultrasound images from three tumour types across three stages of surgery.

A total of nine anonymized representative iUS images were captured from three procedures (Figure 1). The GE healthcare bk5000 ultrasound device was utilized. The iUS images were optimised by the operating surgeon (NB) across 3 parameters: frequency (5–13 MHz), depth (10–80 mm) and gain. The images included a histologically confirmed high grade glioma (HGG), low grade glioma (LGG) and brain metastasis. We chose the commonest intra-axial tumours treated surgically in our department with a known span of margin ambiguity. During each procedure, images were captured at three stages: before tumour resection, during resection and near completion of resection. A total of 18 neurosurgeons participated in the study. First, participants completed a survey detailing their grade (attending, fellow or resident) and their experience with using intra-operative ultrasonography for brain tumour surgery (<10 cases; 10–25 cases; 25–50 cases or >50 cases). Using 3D Slicer, participants were invited to draw around (segment) what they believed was tumour in the nine images. Participants were supported with the use of 3D Slicer by members of the research team (AJ, JW, OS). Participants were free to decide how many areas to segment, including none. For each image, the participant also rated how difficult they found the segmentation on a Likert scale from 1 (very easy) to 10 (very hard). Ethical approval was waived for this study as it used anonymised images captured as part of routine practice and involved voluntary participation by neurosurgeons.

### Statistical analysis

Using Python through the ChatGPT Data Analyst interface, segmentations were converted into binary masks and then analysed using the Digital Image Correlation Engine Similarity Coefficient (DSC) which measures the similarity between two images by comparing pixel patterns (12). DSC range from 0 to 1 with segmentations that are perfectly aligned getting a score of 1 and segmentations that are not aligned at all getting a score of 0. For each image, every observer was compared against the other, producing a DSC for each comparison. In this study, a total of 153 DSCs were calculated for each image across the 18 participants. Heat maps were created for each image by converting the binary masks into arrays and allocating each pixel an aggregate score based upon whether it was incorporated within the segmented tumour. Each pixel could have a score ranging from 0 (all participants deemed this pixel not to be tumour) to 18 (all participants deemed this pixel to be tumour). The following colour code was then allocated to the pixels based on their aggregate score: green (15–18), red (4–14) and white (0–4). This heatmap was made transparent and then overlaid onto the original iUS image. Comparisons between the stages of surgery and tumour type were performed with the Kruskal–Wallis test with Dunn's multiple comparisons test. Correlation between the average difficulty ratings and DSCs for the 9 iUS images was performed using Spearman's correlation. Comparisons between high and low experience surgeons across tumour type and operation stage was performed using two-way ANOVA with Tukey multiple comparisons test. Statistical analysis and graphical visualisation were conducted using R through the BioRender interface.

## Results

**Figure 2:**
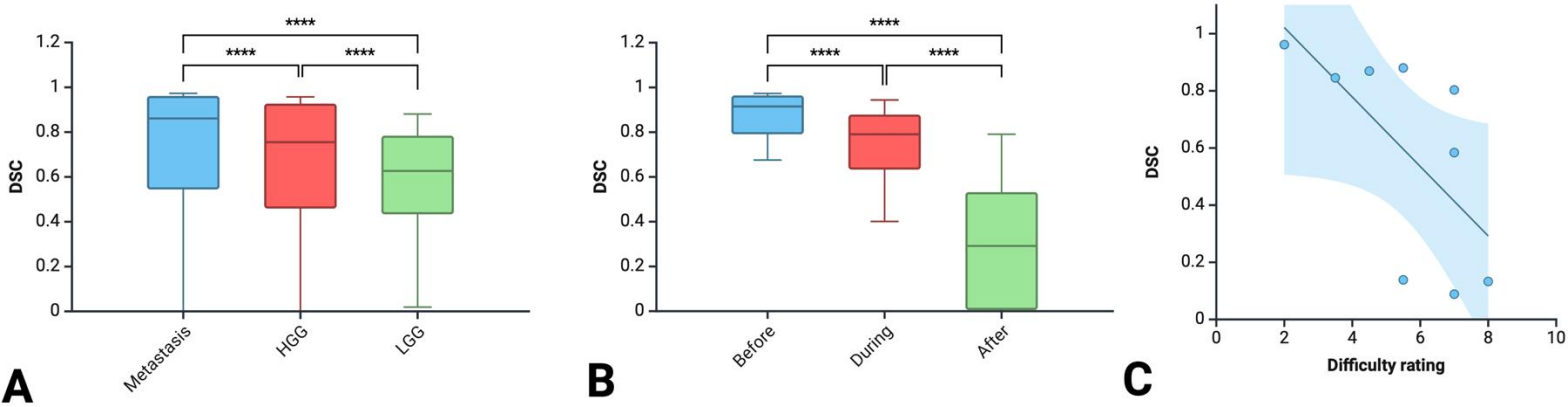
Comparison of DSC between the three tumour types **(A)** between the three stages of surgery **(B)** and correlation between DSC and difficulty rating **(C)**.

**Figure 3:**
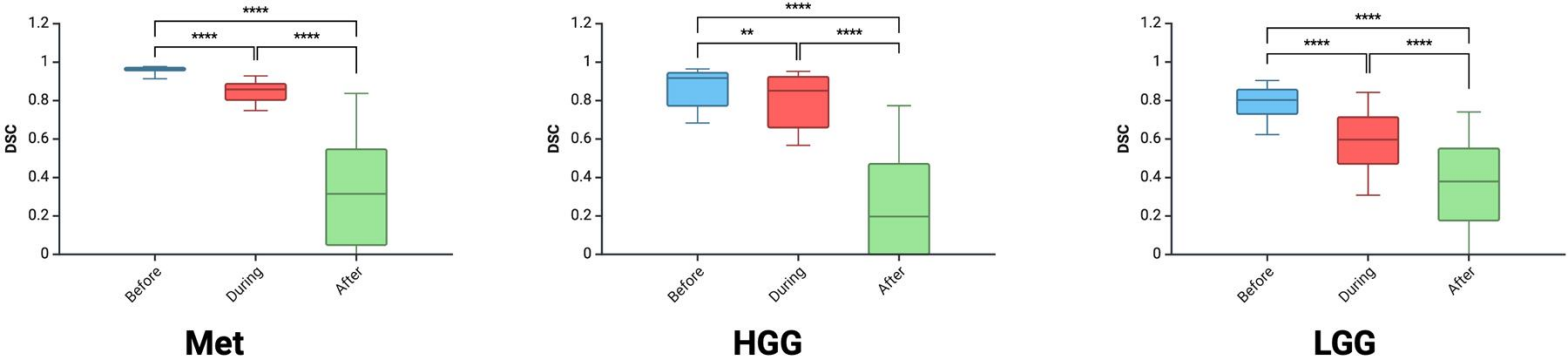
Tumour-specific comparison of DSC across three stages of surgery.

**Figure 4:**
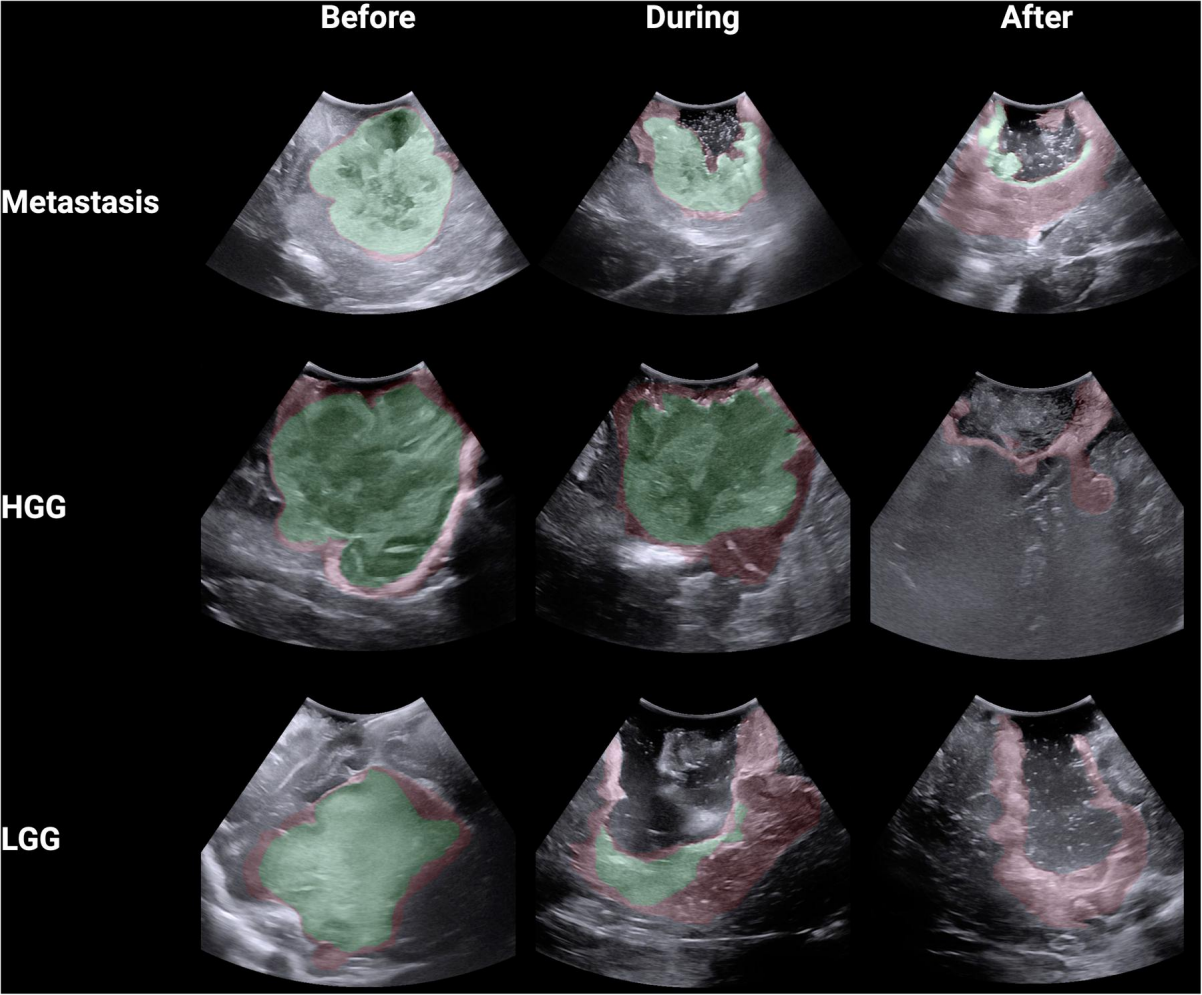
Spatial heat maps of observer agreement across 9 iUS images (green >80% agreement; red 20%–79% agreement).

A total of 18 neurosurgeons participated in the study including 5 attendings, 7 fellows and 6 residents. From this group, a total of 1,377 DSCs were calculated across the 9 iUS images. Metastasis had the highest DSC (0.72 ± 0.32), followed by HGG (0.64 ± 0.33) and then LGG (0.58 ± 0.25; *p* < 0.00001) (Figure 2A). As the resection progressed, the degree of inter-observer agreement broke down. Before resection the DSC was 0.871 ± 0.105; during resection (0.74 ± 0.174) and at completion (0.32 ± 0.270; *p* < 0.00001) (Figure 2B). Observers reported increasing difficulty with iUS interpretation as the resection proceeded and there was statistically significant (*p* = 0.014) negative correlation (−0.775) between DSC and difficulty rating of the segmentation (Figure 2C). The trend of decreasing agreement as the resection progressed held across tumour types. It was most pronounced in HGG where there was a near complete breakdown of agreement in the resection cavity (0.26 ± 0.28) (Figure 3). Heats maps for the iUS demonstrated the spatial variation in observer agreement across the tumour types and stages of surgery (Figure 4). It shows decreasing agreement as the resection proceeds particularly within the margins of the resection cavity near the end of the resection. From the 18 participants, there were 5 observes who were highly experienced with iUS (>50 cases) and 6 observers with low experience (<10 cases). There was no significant difference in DSCs between the high and low experience observers across the tumour types and stages of surgery.

## Discussion

In this study, we report the an assessment of inter-observer agreement in interpreting iUS images from brain tumour surgery. We found that inter-observer agreement was both tumour-type and operation-stage dependent. Agreement was strongest for brain metastases and then diminished for diffuse primary brain tumours. Also, agreement started strong before the surgical resection commenced but broke down as the surgery proceeded. When we conducted 2D spatial analysis, we found that the breakdown in agreement was most marked at the base of the resection cavity. Extent of resection is an important factor in improving survival in patients with brain tumours (17, 18). A range of technologies have been introduced to support neurosurgeons to enhance the extent of resection during brain tumour surgery including fluorescence-guided surgery, intra-operative MRI and optical neuro-navigation (13). Ultrasonography is part of this armamentarium and there is evidence that its use can improve extent of resection (2, 3). However, iUS is user dependent as it requires real-time subjective interpretation of images. This is particularly relevant in brain tumour surgery, as interpretation of the surgical cavity margins is important in supporting intra-operative decision-making to extend the resection or not.

### Inter-observer agreement in ultrasonography

Broadly, there is good inter-observer agreement in the use of ultrasonography in medicine (5, 14, 15). However, this agreement can be variable and starts to breakdown for certain measurements and assessments. A study examining interpretation of thyroid nodule ultrasound images found that there was strong agreement on determining nodule calcification and vascularity but moderate agreement on the nodule shapes and margins (5). This variability in agreement based on the assessed parameters is important, particularly in the context of iUS for brain tumour surgery. Given the importance of extent of resection and the interpretation of tumour margins, our study was specifically designed to probe the question of agreement at the resection cavity margins. Interpreting these images is notoriously difficult due to the phenomenon known as posterior wall acoustic enhancement (PAE) (10). PAE often appears beneath fluid-filled structures like cysts as fluid attenuates ultrasound less than solid tissue, creating stronger echoes and higher echogenicity. This can complicate differentiation between residual tumour and PAE at the base of resection cavities. Chacko and colleagues took samples from resection cavities and found the sensitivity of iUS in detecting tumour at the tumour-brain interface was 97.1%, however its specificity was lower at 53.6% (16). The high false positive rate is likely related to PAE and hyperechoic clot. A novel approach to minimise PAE is to use a coupling fluid designed to have an attenuation coefficient similar to brain tissue which helps reduce the enhancement artifacts caused by saline solutions typically used in resection cavities (19). The difference in DSC between tumour types is interesting and relates to differing echogenic signal of each tumour. Metastases and HGG usually appear more hyperechoic, irregular, and well-circumscribed on iUS, often with hypoechoic necrotic areas and hyperechoic oedema, whereas LGG are typically iso- to mildly hyperechoic with less distinct margin (10). This distinction makes it easier to segment metastases and HGG compared to LGG which is supported by our study.

### Role of experience in inter-observer agreement

The experience of observers has been shown to improve inter-observer agreement in MRI (20). The theory behind this is that experienced observers coalesce around an accurate diagnosis compared to less experienced observers where is a higher chance of random or incorrect interpretations. Our study found that there was no significant difference between the observers based on their experience. The ultrasound literature has shown conflicting findings on the impact of experience on inter-observer agreement. A study examining transvaginal ultrasound for local staging of cervical cancer found that experience improved observer agreement for only one of three measured metrics (21). Conversely, the Swiss Sonography in Arthritis and Rheumatism (SONAR) group developed a semi-quantitative score for synovitis and erosion in Rheumatoid Arthritis and found that the experience of the sonographer substantially improved agreement (22). The explanation for the variability in these findings may be related to the highly user dependent nature of ultrasound. Given raters would need to position the ultrasound probe to generate the image for interpretation, this adds a further layer of variability which may impact the degree of agreement. This may provide an explanation for why we did not identify differences between low and high experience observers. Experienced observers would manipulate the ultrasound probe in a way that helps them identify residual tumour and this important facet of experience is lost when using still images which are more open to random interpretation.

### Future directions

Artificial intelligence is playing an increasing large role in neurosurgery. This spans improving surgical workflows, real-time monitoring and diagnosis, outcome prediction, volumetric assessment, and neurosurgical education (23–25). One approach to address this agreement gap in iUS, is to take a quantitative approach to interpreting the ultrasound images. Basic image analysis techniques for iUS for brain tumour surgery showed that pixel brightness correlated with histological features (26). More recently, machine learning approaches have been utilised for both real-time tumour differentiation and histological and molecular diagnosis (27–29). Cepeda and colleagues characterized quantitative texture analysis in B-mode and elastography which was found to be significantly associated with overall survival (30). More recently, a multicentre study using the brain tumour intraoperative ultrasound database (BraTioUS) demonstrated the feasibility of a convolutional neural network (CNN) model for glioma segmentation (31).

### Limitations

The study had several limitations. The main limitation was that we did not have a ground truth to compare the observers' segmentations to. This could have included histological biopsies that were correlated to the iUS images to help determine what was truly tumour and what was not in the image. Secondly, the experimental design differed from routine practice with static images presented to the observer to interpret. This is different to real life situations where surgeons can move the probe to get real-time feedback which can help with interpreting the iUS images. Finally, our study included 9 images which is less compared to other studies in the literature (11). We optimised the study to maximise the number of observers and collected responses from 18 neurosurgeons which is a major strength of the study.

## Conclusions

This study demonstrates that inter-observer agreement in interpreting iUS images during brain tumour surgery varies significantly by tumour type and resection stage. The findings underscore the challenges of accurately interpreting tumour margins, particularly in the resection cavity. While experience did not significantly impact agreement, the study highlights the need for quantitative approaches using machine learning to improve consistency and accuracy in iUS interpretation for brain tumour surgery.

## Data Availability

The raw data supporting the conclusions of this article will be made available by the authors, without undue reservation.
